# Evaluating real-time internet therapy and online self-help for problematic alcohol consumers: a three-arm RCT protocol

**DOI:** 10.1186/1471-2458-9-16

**Published:** 2009-01-14

**Authors:** Matthijs Blankers, Maarten Koeter, Gerard M Schippers

**Affiliations:** 1 Amsterdam Institute for Addiction Research (AIAR), Academic Medical Center, University of Amsterdam, Department of Psychiatry, Room PB 0.425, PO Box 75867, 1070 AW Amsterdam, the Netherlands; 2 Amsterdam Institute for Addiction Research (AIAR), Academic Medical Center, University of Amsterdam, Department of Psychiatry, Room PA 1.181, PO Box 75867, 1070 AW Amsterdam, the Netherlands; 3 Amsterdam Institute for Addiction Research (AIAR), Academic Medical Center, University of Amsterdam, Department of Psychiatry, Room PA 1.190, PO Box 75867, 1070 AW Amsterdam, the Netherlands

## Abstract

**Background:**

Only a minority of all alcohol- and drug abusers is receiving professional care. In an attempt to narrow this treatment gap, treatment facilities experiment with online healthcare. Therefore, it is important to test the (cost-)effectiveness of online health interventions in a randomized clinical trial.

**Methods:**

This paper presents the protocol of a three-arm randomized clinical trial to test the (cost-) effectiveness of online treatment for problem drinkers. Self-help online, therapy online and a waiting list are tested against each other. Primary outcome is change in alcohol consumption. Secondary outcome measures include quality of life and working ability. Incremental cost-effectiveness ratios for self-help online alcohol and therapy online alcohol will be calculated. The predictive validity of participant characteristics on treatment adherence and outcome will be explored.

**Discussion:**

To our best knowledge, this randomized clinical trial will be the first to test the effectiveness of therapy online against both self-help online and a waiting-list. It will provide evidence on (cost-) effectiveness of online treatment for problem drinkers and investigate outcome predictors.

**Trial registration:**

This trial is registered in the Dutch Trialregister (Cochrane Collaboration) and traceable as NTR-TC1155.

## Background

The Netherlands have, compared to most other countries, a fairly accessible network of substance abuse treatment centres (SATCs). However, the target population of these SATCs is not sufficiently addressed: Only a minority of all current alcohol- and drug abusers is receiving professional care [1]. This so-called treatment gap is mainly due to two reasons. As substance dependence is strongly stigmatized, the threshold to visit a treatment centre is high [2]. And as most SATCs offer outpatient facilities during working hours only, many people with jobs cannot find to attend SATCs face-to-face therapy.

Another challenge to healthcare in general and addiction treatment in particular is optimising cost-effectiveness. As the future health spending is expected to increase at a much higher rate than in the past [3,4], cost-effectiveness is an important parameter in the development of new treatment programs. In general, early interventions, preventive care, and disease self-management, together with the correct implementation of health information technology and internet applications, is believed to have the potential to enhance health status while improving efficiency and reducing healthcare costs [3].

Substance abuse treatment over the internet has the potential to address these issues: The influence of both stigmatization and restricted opening times of healthcare institutions is reduced when clients do not have to visit a SATC during working hours, but can visit their web-based treatment environment from any place and at any time. Earlier evaluations have shown that a majority of online substance abuse treatment participants manage to hold their jobs [5,6]. Therefore, improvement in job performance may be expected as a result of successful participation, as problematic drinking habits tend to interfere with working ability in general.

The increasing number of online treatment options worldwide shows that treatment facilities are willing to experiment with online healthcare. A review of the current state of the art in substance abuse treatment over the internet is promising [7]. Some authors report online substance abuse treatment to be successful in addressing an underserved population [5,6]. Results of the first randomized clinical trials support the use of the internet to extend the treatment options for substance abusers [8-10]. There is preliminary evidence on the cost-effectiveness of early interventions and computer-aided therapy for alcohol related problems [11,12]. However, as the number of randomized clinical trials for online substance abuse treatment is still sparse, its (cost-)effectiveness is yet to be supported more firmly. This article presents a randomized clinical trial that will extend the knowledge on effectiveness and cost-effectiveness of online treatment for problem drinkers.

The SATC whose interventions are subjected to this trial has pioneered in the development of internet applications for problematic alcohol and substance users. Since 2003, its online treatment programs have been based on two evidence-based techniques: cognitive-behavioural therapy (CBT) and motivational enhancement training. These early experiences have led to the development of online interventions for alcohol-, cannabis-, cocaine- and tobacco-users, and for pathological gamblers. Two online treatment programs for problem drinkers will be compared in this trial. One is an anonymous, online, non-counsellor involved, fully automated self-guided treatment program, referred to as Self-help Online Alcohol (SOA). The other is a real time, online, non anonymous, counsellor-guided therapy program for problem drinkers referred to as Therapy Online Alcohol (TOA).

SOA introduces participants to various CBT elements as a means to monitor and help them change their alcohol consumption. Registration of alcohol consumption, online diary, goal setting, and relapse prevention are among them. A recent study has shown that SOA is highly attractive and promising in its effects [6]. However, participants of SOA did report they missed the opportunity to interact with a counsellor on a regular basis. In response to the participants' suggestions, the online therapy program (TOA) has been developed. Like SOA, TOA is based on CBT and motivational enhancement training, but has a more intensive and stricter structured treatment course and regular counsellor interaction. Contact between client and counsellor takes place online in a one-on-one, private chat-room. TOA resembles to a great extent the conventional face-to-face manual based CBT training program [13], used in many SATCs in the Netherlands. Treatment elements of TOA include consumption registration, online diary, tailored feedback of recent alcohol consumption, contemplating advantages and disadvantages of drinking, goal setting, self-control methods, investigating risk-situations, coping with craving, emotions and social pressure, and the formulation of emergency plans in case of relapse. These elements are introduced by the counsellor. Working with the treatment elements is part of clients' weekly homework assignments.

The most important difference between SOA and TOA are seven real-time, one-on-one chat sessions with an experienced counsellor trained in TOA. Each chat session lasts for approximately 40 minutes. During these sessions, feedback on homework assignments is provided and the client is motivated to enhance his or her attempt to stop or reduce drinking. Because of it's interactive nature, chat-sessions resemble to a big extent the conversational dynamics of regular, face-to-face therapy contacts. This new element meets the expressed needs of participants of SOA and makes the intervention more similar to the successful face-to-face CBT treatment.

## Methods

### Aims of the trial

This study aims to test the effectiveness and cost-effectiveness of SOA and TOA against a waiting list control group, in a three-arm randomized clinical trial. Primary outcome measure is the change in alcohol consumption from baseline (t0) to the three month follow-up (t1) and to the six month follow-up (t2). Secondary outcome measures are changes in AUDIT scores, quality of life and quality of functioning at work. Incremental cost-effectiveness ratios for SOA and TOA will be calculated. The predictive validity of participant characteristics on treatment adherence and outcome will be explored.

### Study population

Our study population will be recruited through the SATCs website, that was viewed by 650.000 unique visitors in 2007 [14]. If necessary, additional attention for this study will be attracted through advertisements at family doctors' offices, internet-forums, and news-paper adds. Applicants meeting inclusion criteria defined in Table 1 are invited to participate.

**Table 1:** Selection criteria for inclusion

**Inclusion criteria**	**Exclusion criteria**
age 18 – 64	prior therapy in addiction treatment centre
internet at home	history of alcohol delirium
AUDIT score > 8	history of drug overdose
alcohol consumption > 14 standard units per week	severe coronary problems
informed consent received	severe intestine diseases
	diagnosed schizophrenic
	history of stroke
	epilepsy in last year
	contemplated or attempted suicide in last year
	cocaine and or amphetamine use > 4 days last month
	cannabis use > 9 days last month
	holiday/travelling intention > 2 weeks during trial

### Research questions

Q1a. Do TOA participants reduce their alcohol consumption from t0 to t1 and t2 to a larger extent than SOA and waiting list control participants?

Q1b. Do TOA participants improve to a larger extent on secondary outcome measurement scores from t0 to t1 and t2 than SOA and waiting list control participants?

Q1c. Are TOA participants more often treatment-responders than SOA and waiting list control participants at t1 and t2?

Q1d. Do SOA participants reduce their alcohol consumption to a larger extent from t0 to t1 and t2 than waiting list control participants?

Q1e. Do SOA participants improve on secondary outcome measurement scores from t0 to t1 and t2 to a larger extent than waiting list control participants?

Q1f. Are SOA participants more often treatment-responders than waiting list control participants at t1 and t2?

Q2. What are the comparative costs per quality adjusted life year (QALY) for SOA and TOA?

Q3a. How do participants' demographic characteristics relate to demographics of participants in conventional face-to-face CBT programs?

Q3b. Do scores of working alliance between TOA participants and counsellor predict treatment-response at t1 and t2?

### Hypotheses

We hypothesize TOA participants will have reduced their alcohol consumption significantly more at the three month follow-up (t1) compared to SOA and waiting list control participants (Q1a). Between t0 and t1, we also expect TOA participants to improve on secondary outcome measurements (quality of life and working abilities) significantly more than SOA and waiting list control participants (Q1b). We hypothesize that a larger percentage of TOA participants will meet the criteria for treatment-responders compared to SOA and waiting list control participants (Q1c). We hypothesize that SOA participants will have reduced their t0 alcohol consumption significantly more at t1 than waiting list control participants (Q1d). Between t0 and t1, we also expect SOA participants to improve on secondary outcome measurements significantly more than waiting list control participants (Q1e). Finally, we hypothesize that a larger percentage of SOA participants will meet the criteria for treatment-responders compared to waiting list control participants (Q1f).

Between t1 and t2, we expect TOA participants to be better able than SOA participants to maintain their achieved changes in alcohol consumption, resulting in a significant time (t1–t2) by group (SOA-TOA) interaction. Alcohol consumption in SOA will be significantly higher at t2 compared to t1, whereas alcohol consumption in TOA will not change significantly during this period.

For question 2, we hypothesize that from t0 to t1 both the costs and the gains in QALY for SOA to be lower than the costs and the gains in QALY for TOA. As this means that both the numerator and the denominator of the cost-effectiveness equation is larger for TOA compared to SOA, we have no preconceptions of whether the incremental cost-effectiveness calculation will favour either SOA or TOA (Q2).

For question 3, we hypothesize our participants to be higher educated, a larger percentage to be working and female, compared to conventional outpatient SATC clients (Q3a). We also hypothesize that higher scores on working alliance will predict a larger proportion of treatment-response at t1 for TOA participants (Q3b).

### Measurement instruments

Several measurement instruments are included in the t0, t1 and t2 questionnaire, as presented in Table 2. Alcohol consumption will be measured using substance use subscales of the 'European Addiction Severity Index' (EuropASI) [15] and Timeline-Follow-Back for alcohol consumption [16]. This Timeline-Follow-Back method has good capacity to retrieve drinking history in the past week [17]. As a third scale to measure alcohol consumption burden the AUDIT is included [18].

**Table 2:** Measurement instruments

**Instrument**	**t0 measure**	**t1 measure**	**t2 measure**
EuropASI (subscales)	x	x	x
Timeline-follow-back	x	x	x
AUDIT	x	x	x
QOL	x	x	x
EQ-5D	x	x	x
WAI-work	x	x	x
SF-HLQ	x	x	x
BSI	x	x	x
MCSDS	x		
Dutch mental health thermometer		x*	x*
WAI		x*	x*

Quality of life will be measured using two instruments: The EQ-5D [19-21] and the QOL [22]. The EQ-5D is a highly accepted, widely used and easy to administer instrument for measuring quality of life for alcohol and drug dependents [23,24]. As the scoring algorithm of the EQ-5D generates an index weighted by a preference measure, EQ-5D scores can be used in cost-effectiveness analysis to estimate QALYs [25]. However, it is recommended that the EQ-5D is used in combination with other quality of life instruments. This should be done to minimize its potential limitation in sensitivity until further results confirm EQ-5D validity in alcohol dependent individuals [26]. Therefore, the 'Quality Of Life' scale QOL [22] is included. According to content validity analysis, this is a valid instrument for measuring quality of life across patient groups and cultures. It is conceptually distinct from health status or other causal indicators of quality of life [27].

As an earlier evaluation study of SOA has shown that a majority of its participants manage to hold jobs [5,6], their problematic drinking habit could negatively influence their working ability. To be able to measure this effect, we included two working ability measurement scales. The SF-HLQ, a subscale of the Trimbos/iMTA questionnaire for Costs associated with Psychiatric Illness (TiC-P) [28], is well able to measure the work related productivity of applicants who held a job in the past two weeks. A more general measure of working ability, which is also eks held a job. A more general applicable for the applicants who are not currently employed, is provided by the 'Working Ability Index' (WAI-work) [29]. This questionnaire measures self-reported ability to work, as opposed to the SF-HLQ which measures the actual working behaviour.

Key demographical characteristics will be measured using subscales of the EuropASI [15]. Baseline psychopathology will be measured using the Brief Symptom Inventory (BSI) [30,31]. To be able to evaluate the reliability of the self-reported questionnaire data, a 13-item version of the Marlowe Crowne Social Desirability Scale (MCSDS) [32] is included.

### Primary and secondary outcome measures

Primary outcome measures are the differences in alcohol consumption, and the number of participants showing 'treatment response', as defined below. Although de facto a composite score of primary and secondary outcomes, 'treatment response' is considered primary outcome as well. Secondary outcome measurements are AUDIT-score; quality of life as measured by QOL, and EQ-5D, psychopathological symptoms as measured by BSI and ability to work as measured by SF-HLQ and WAI-work.

### Definition of treatment-response

Adding treatment-response criteria has an important advantage over the comparison of group mean-scores on primary and secondary outcome measures only. In our opinion, it has more external validity as it is helpful in making a clear statement on who actually improved to a clinical relevant extent. Based on binary treatment-response outcome data, we can calculate relative risk reduction, number needed to treat, and odds ratios. The British Medical Association published guidelines on sensible drinking which suggested that the boundaries for sensible alcohol consumption should be set at a maximum of 21 standard alcohol units per week for men, and 14 standard alcohol units per week for women [33]. To calculate treatment-response outcome, alcohol consumption as measured using Timeline-Follow-Back will be used [16]. Deteriation percentages relate to changes within participants on the secondary outcome measurement scores between t0 and t1. Treatment-response is defined as: *'alcohol consumption within British Medical Association boundaries, and less than 10% deteriation on any of the secondary outcome measures'*.

### Power calculation

Appropriate sample size is estimated using Gpower v.3.0.5 software [34]. This calculation is based on the primary outcome measure (alcohol consumption), in a one-way ANOVA test design. Based on [10] we expect an effect-size of *d *= 0.40 when comparing waiting list control and treatment groups. To be able to detect even smaller differential effects (*d *= 0.25) between SOA en TOA with *α *= .05 and *β *= .20, we aim to include 150 participants. The power calculation is summarized in Table 3.

**Table 3:** Power analysis

F tests – ANOVA: Fixed effects, omnibus, one-way		
Analysis:	A priori: Compute required sample size	
Input:	Effect size f	0.25
	α error probability	0.05
	Power (1-β error probability)	0.80
	Number of groups	3
Output:	Non-centrality parameter λ	9.938
	Critical F	3.054
	Numerator df	2
	Denominator df	156
	Total sample size	159
	Actual power	0.805

### Randomization

After the t0 measurement is completed, participants are allocated to one of the three trial arms. Because this randomized clinical trial is internet-based, we will organize the allocation procedures online as well. As the number of participants we aim to include in each trial arm (*n *= 50) is not very large, random variation in baseline characteristics could reduce group equivalence. Online, server-based performance of randomization procedures comes with the advantage of being able to implement stratified allocation or allocation through adaptive randomization relatively easy.

To prevent between-group variability on relevant baseline characteristics, we use an adaptive or biased-coin randomization algorithm for this trial. Adaptive randomization aims to ensure trial arms are balanced with respect to a priori defined participant factors as well as for the number of participants in each group. Simulation studies show that adaptive randomization provides better balanced trial arms when compared with 'normal' randomization [35] and that it is more efficient in balancing multiple poli-level factors than permutated block stratification [36]. Although not widely used due to organizational complexity, adaptive randomization is a highly effective allocation method, and its wider adaptation in the conduct of randomized clinical trials is recommended [37]. The use of adaptive randomization is supported in the CONSORT statement [38]. We will implement the Pocock & Simon Range Method of adaptive randomization [39], with D = 'range', G is the sum of D (no factor weighting), and P = 0.60.

### Procedures

For the purpose of this trial we will develop a website to inform potential applicants about the contents of the online treatment, and the procedures associated with this trial. Upon visiting the website, new participants can read all relevant information and ask additional questions through a mail form. For included participants, a secured login system to enter the online interventions is developed and available on the website. Visitors willing to participate fill out a web-based questionnaire to check if they meet the inclusion criteria. If they do not, they are informed so, and an email is sent to this applicant. This email contains additional information about alternative treatment options. Applicants meeting inclusion criteria proceed to the subscription screen. Next, they are once again informed on the conditions of participation in this trial. After reading all information, they decide whether they are willing to participate. If they would like to participate, they are asked for necessary personal information. A confirmation email is sent to them, with the informed consent as an attachment. This confirmation email also contains their personal username and password. With this unique username/password combination, applicants can log in to their private area. Upon logging in for the first time, applicants are shown a screen which requests them to confirm they read, understood and agreed with the informed consent sent to them. Applicants can decide to agree with this informed consent at any time, and can deliberate on whether or not they consent with the participant information for as long as they want. Only after their confirmation they are introduced to the baseline measurement procedure. After completion of the baseline measurement the randomization procedure takes place. Depending on the outcome, participants are allocated to one of three trial arms.

### Trial flow

**Figure 1:**
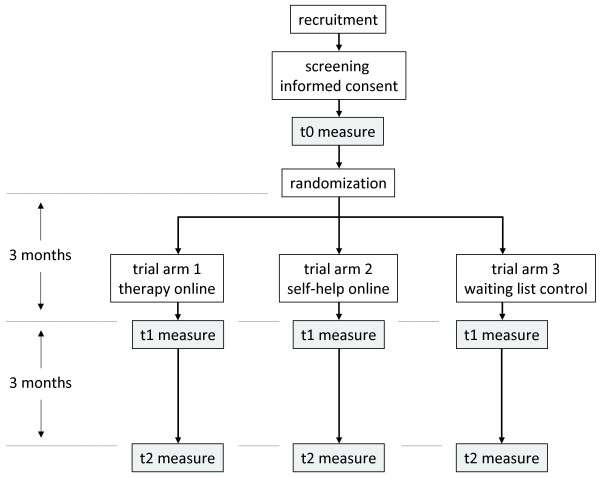
**Trial flowchart**. This figure provides an overview of the participant flow for this trial.

Figure 1 provides an overview of the trial flow. If a participant is allocated to trial arm 1, he or she is directly introduced to TOA through a welcome text. A notification will now be sent to the TOA coordinator to make a first appointment for a chat session with the participant. From now on, TOA starts for participants in trial arm 1. Three months after t0 measurement, participants will receive an invitation for the t1 measurement by email and upon visiting the trial website. It is possible that some participants have not yet finished the entire TOA process after this period. However, the decision has been made to let follow-up timing depend solely on the time interval since t0, not on the current status of their treatment proceedings. This is in our opinion the best possible confirmation to the 'intention to treat' principle [40]. Three months after the t1 measurement participants are invited for the t2 measurement. After completion of t2 measurements, data collection is completed. Participants in trial arm 2 are introduced to SOA after randomization. Up until t1, participants can use the SOA program every time they log in on the trial website. t1 and t2 measurements are introduced by email and upon visiting the trial website. Participants in this trial arm are offered the possibility to proceed with TOA after completion of t2 measurements, would they be interested in more help after completion of SOA. Participants in trial arm 3 are allocated to the waiting list after randomization. After three months, they too will receive an invitation for the t1 measurement. After t1, participants in trial arm 3 are introduced to TOA. For ethical reasons, the decision is made to offer them TOA after three months (t1), and not after t2 (six months).

### Maximize response rates

Drop-out is a potential threat in every clinical trial. Since attrition rates for online trials tend to be larger than in regular ones [52], we will make extra efforts to maximize response rates. Following suggestions for successful strategies to maximize response rates [41,42], we made follow-up questionnaires as short as possible and give participants feedback on the actual length and their proceedings while filling out the questionnaire. We motivate participants to fill out the questionnaires by sending them a reminder if they do not respond within five days after the first emailed invitation. To optimize a feeling of worthiness and to reward participants for their efforts and time, participants will receive gift coupons (€15,-) after filling out a follow-up questionnaire. Participants will be aware of this incentive upon reading the informed consent form. However, participants who do not fill out the questionnaires after a reminder is sent will be contacted by a call centre. Since 2005, this call centre has been collecting regular outcome data of outpatient addiction treatment for SATCs nationwide in the Netherlands. It is experienced in maximizing response rates in hard to reach populations [43]. For this research project, they will collect outcome data by phone from trial participants who do not respond to emailed invitations. All participants are aware of these procedures, provide phone numbers, and consent with receiving phone-calls for follow-up measurements.

### Data analysis

Multiple imputation missing data handling procedures will be implemented using MICE [45] a package for the R Statistical Software Environment. As the quantity of consumed alcoholic drinks is the primary outcome variable in this trial, analysis techniques are chosen that suit the expected data distribution. Baseline measurements of the three trial arms will be compared using t-tests and χ^2^-tests. Trial arm differences on primary and secondary outcome variables at three months are tested using repeated measures ANOVA. Trial arm differences for SOA and TOA between t1 and t2 are tested with a 2 (group) × 2 (time) repeated measures ANCOVA with between and within contrasts. Effect-sizes will be calculated using Cohen's *d *[46]. Trial arm differences on treatment-response will be tested using multiple logistic regression analysis. Costs from TOA and SOA will be derived from cost records of the collaborating SATC. Costs will be assessed in correspondence to the recommendations by the U.S. Panel on Cost-effectiveness in Health and Medicine [25], following a macro-costing approach. QALYs will be calculated from the EQ-5D questionnaire, using algorithm by Dolan [47], retrieved from the Open Health Measures website [48]. Based on these estimations, costs-per-QALY and an incremental cost-effectiveness ratio can be calculated. The U.S. Panel on Cost-effectiveness has recommended that cost-effectiveness studies should conduct sensitivity analysis, which for this cost-effectiveness analysis will be implemented through the bootstrap method [49-51].

### Ethical approval

This trial will be executed in compliance to the Helsinki Declaration and is approved by the Medical Ethical Commission of the Academic Medical Center in Amsterdam, the Netherlands.

## Discussion

To our best knowledge, this randomized clinical trial is the first to test the effectiveness of online therapy (TOA) against both a derived self-help program (SOA) and a waiting-list control group. Both SOA and TOA are based on an evidence-based CBT face-to-face treatment programme [13], implemented by several SATCs in the Netherlands. The counsellors involved in TOA are all working for a well respected, research minded SATC with many years of experience in the counselling of problem drinkers. The AIAR (Amsterdam Institute for Addiction Research) group has over 15 years of experience in both clinical and experimental addiction research. This solid foundation makes a sound opportunity to design and execute a randomized clinical trial to test effectiveness, while on the other hand extending knowledge on how internet technology can be used in healthcare research.

A potential threat for this trial is the so-called 'law of attrition', defined by Eysenbach: 'the phenomenon of participants stopping usage and/or being lost to follow-up, as one of the fundamental characteristics and methodological challenges in the evaluation of eHealth applications' [52]. We intent to implement four measures to reduce the impact of this attrition law. First, data-collection over the telephone, performed by an experienced call centre as a backup method for the follow-up measurements gives us a greater possibility to actively pursuit higher response rates. Second, all participants in this trial have to make a major time investment before being randomized and included in this trial – by filling out a 45 minutes baseline measurement. As an extra incentive for participation to the follow-up measurements, all participants receive gift coupons for their filled-out questionnaires. As a last resolve, all missing data values in the final data set will be multiple imputed according to methodology suggested by Schafer and Graham (2002) [44].

A second potential risk in this trial would concern the inclusion of participants. The inclusion of enough participants is not our main sorrow. As SOA in the regular context and without promotional efforts is able to attract over 150 new participants each month [6], we think we will manage to include 150 participants in a one year inclusion period. However, the resemblance of the regular online clientele is our main focus of attention. Due to the high accessibility of online help in the normal context and the relatively high constraints posed on participants in this trial, there is a potential risk that the trial participants do not resemble the regular e-clientele exactly. Whether this actually occurs or not can only be answered afterwards, by comparing data on characteristics of trial participants and regular online clientele data.

A third possible limitation concerns integrity of our collected data. As our t0 and t1/t2 data is self reported without a researcher nearby to oversee data-collection, we rely heavily on the correctness of our participants' answers. Various authors evaluate the self-reported data in alcohol research as reliable [53-55]. Tourangeau and colleagues even concluded that self-reported data is more reliable in the absence of a research confederate [56]. However, as an extra means to check data integrity, we included a social desirability scale, to measure whether these tendencies occur in our research population.

Another point of concern on data integrity in this trial could be made about our suggested statistical procedures. Multivariate statistics relies considerably on data distribution normality [57]. As the distribution of alcohol consumption count data is usually zero-elevated and highly skewed, particularly in subjects with alcohol dependence [58], multivariate statistics and most statistical tests should be used with caution. To prevent possible in- or deflated α's, we will use bootstrap methods and permutation tests as suggested by Hesterberg and colleagues [59], to minimize the influence of data distributions on our testing results, if data turn out to be non-normally distributed.

During the planning stage of this trial we have thought of implementing a conventional face-to-face treatment comparison condition as well. However, it is disputable whether in clinical practice, the face-to-face treatment and the online treatment populations are comparable. In previous pilot studies, we found that less than 10% of the internet treatment population was willing to receive conventional treatment [6]. On the other hand, all face-to-face participants who consider themselves as computer-illiterate, or do not have a computer with internet connection at home, would be unsuitable for online treatment. These considerations illustrate not only the methodological difficulties of a hybrid online – face-to-face trial, but also the restricted external validity of it's results. This was for us the main reason not to chose for a conventional – internet therapy comparison.

## Trial registration

This trial is registered in the Dutch Trialregister (Cochrane Collaboration) and traceable as NTR-TC1155.

## Competing interests

The authors declare that they have no competing interests.

## Authors' contributions

All authors have contributed substantially to this protocol. MB constructed the design of the study and drafted the manuscript. MK led on the overall methodological development and revised the manuscript. GMS is principal investigator, constructed the design of the study and drafted the manuscript. All authors have read and approved the final manuscript.

## Pre-publication history

The pre-publication history for this paper can be accessed here:



## References

[B1] Bijl RV, van Zessen G, Ravelli A (1997). Psychiatrische morbiditeit onder volwassenen in Nederland: het NEMESIS-onderzoek II. Prevalentie van psychiatrische stoornissen. Ned Tijdschr Geneeskd.

[B2] Room R (2005). Stigma, social inequality and alcohol and drug use. Drug Alcohol Rev.

[B3] PriceWaterhouseCoopers' Health Research Institute (2007). HealthCast 2020: creating a sustainable future.

[B4] Ministerie van Volksgezondheid, Welzijn en Sport (2006). Kiezen voor gezond leven 2007–2010.

[B5] Postel M, de Jong C, de Haan H (2005). Does e-therapy for problem drinking reach hidden populations?. Am J Psychiatry.

[B6] Blankers M, Kerssemakers R, Schramade M, Schippers GM (2007). Eerste ervaringen met internet-zelfhulp voor probleemdrinkers. Maandblad Geestelijke volksgezondheid.

[B7] Copeland J, Martin G (2004). Web-based interventions for substance use disorders: a qualitative review. Journal of Subst Abuse Treat.

[B8] Hester RK, Delaney HD (1997). Behavioral self-control program for Windows: results of a controlled clinical trial. J Consult Clin Psychol.

[B9] Kypri K, Saunders JB, Williams SM, McGee RO, Langley JD, Cashell-Smith ML, Gallagher SJ (2004). Web-based screening and brief intervention for hazardous drinking: a double-blind randomized controlled trial. Addiction.

[B10] Riper H, Kramer J, Smit F, Conijn B, Schippers GM, Cuijpers P (2008). Web-based self-help for problem drinkers: a pragmatic randomized trial. Addiction.

[B11] Babor TF, Higgins-Biddle JC, Dauser D, Burleson JA, Zarkin GA, Bray J (2006). Brief interventions for at-risk drinking: patient outcomes and cost-effectiveness in managed care organizations. Alcohol Alcohol.

[B12] McCollister KE, French MT (2003). The relative contribution of outcome domains in the total economic benefit of addiction interventions: a review of first findings. Addiction.

[B13] de Wildt W, Schippers GM, Keijsers G, van Minnen A, Hoogduin CAL (2004). Protocollaire behandeling van patiënten met alcoholproblemen. Motivering, zelfcontroletraining en terugvalpreventie. Protocollaire behandelingen in de ambulante geestelijke gezondheidszorg, II.

[B14] JellinekMentrum (2008). Jaarverslag 2007.

[B15] Kokkevi A, Hartgers C (1995). EuropASI: european adaptation of a multidimensional assessment instrument for drug and alcohol dependence. Eur Addict Res.

[B16] Sobell LC, Sobell MB, Allen J, Litten RZ (1992). Timeline Follow-back: A technique for assessing self-reported ethanol consumption. Measuring alcohol consumption: psychosocial and biological methods.

[B17] Carney MA, Tennen H, Affleck G, Del Boca FK, Kranzler HR (1998). Levels and patterns of alcohol consumption using timeline follow-back, daily diaries, and real-time "electronic interviews". J Stud Alcohol.

[B18] Saunders JB, Aasland OG, Babor TF, de la Fuente JR, Grant M (1993). Development of the alcohol use disorders identification test (AUDIT): WHO collaborative project on early detection of persons with harmful alcohol consumption. Addiction.

[B19] EuroQol Group (1990). EuroQol: a new facility for the measurement of health-related quality of life. Health Policy.

[B20] Rabin R, de Charro F (2001). EQ-5D: a measure of health status from the EuroQol Group. Ann Med.

[B21] Lamers LM, Stalmeier PF, McDonnell J (2005). Measuring the quality of life in economic evaluations: the Dutch EQ-5D tariff. Ned Tijdschr Geneeskd.

[B22] Flanagan JC (1978). A research approach to improving our quality of life. Am Psychol.

[B23] UKATT Research Team (2005). Cost-effectiveness of treatment for alcohol problems: findings of the randomised UK alcohol treatment trial (UKATT). BMJ.

[B24] Zanden BP van der, Dijkgraaf MGW, Blanken P, de Borgie CA, van Ree JM, Brink W van den (2006). Validity of the EQ-5D as a generic health outcome instrument in a heroin-dependent population. Drug Alcohol Depend.

[B25] Petitti DB (2000). Meta-analysis, decision analysis, and cost-effectiveness analysis.

[B26] Günther OH, Roick C, Angermeyer MC, König HH (2008). Responsiveness of EQ-5D utility indices in alcohol-dependent patients. Drug Alcohol Depend.

[B27] Burckhardt CS, Anderson KL (2003). The quality of life scale (QOLS): reliability, validity, and utilization. Health Qual Life Outcomes.

[B28] Hakkaart-van Roijen L, van Straten A, Donker M, Tiemens B (2002). Manual Trimbos/iMTA questionnaire for costs associated with psychiatric illness (TiC-P).

[B29] Tuomi K, Ilmarinen J, Jahkola A, Katajarinne L, Tulkki A (1998). Work Ability Index.

[B30] de Beurs E, Zitman FG (2006). The brief symptom inventory (BSI): the reliability and validity of a brief alternative of the SCL-90. Maandblad Geestelijke Volksgezondheid.

[B31] Derogatis LR, Melisaratos N (1983). The brief symptom inventory: an introductory report. Physol Med.

[B32] Crowne D, Marlowe D (1960). A new scale of social desirability independent of psychopathology. J Consult Clin Psychol.

[B33] British Medical Association (1995). Guidelines on Sensible Drinking.

[B34] Erdfelder E, Faul F, Buchner A (1996). GPOWER: a general power analysis program. Behav Res Methods.

[B35] Rovers MM, Straatman H, Zielhuis GA, Ingels K, Wilt GJ van der (2000). Using a balancing procedure in multicenter clinical trials: simulation of patient allocation based on a trial of ventilation tubes for otitis media with effusion in infants. Int J Technol Assess Health Care.

[B36] Pocock SJ (1979). Allocation of patients to treatment in clinical trials. Biometrics.

[B37] Scott NW, McPherson GC, Ramsay CR, Campbell MK (2002). The method of minimization for allocation to clinical trials: a review. Control Clin Trials.

[B38] Altman DG, Schulz KF, Moher D, Egger M, Davidoff F, Elbourne D, Gøtzsche PC, Lang T (2001). The revised CONSORT statement for reporting randomized trials: explanation and elaboration. Ann Intern Med.

[B39] Pocock SJ, Simon R (1975). Sequential treatment assignment with balancing for prognostic factors in the controlled clinical trial. Biometrics.

[B40] Alderson P, Green S (2002). Cochrane Collaboration open learning material for reviewers.

[B41] Andrews D, Nonnecke B, Preece J (2003). Electronic survey methodology: a case study in reaching hard-to-involve internet users. Int J Hum Comput Interact.

[B42] Kaplowitz MD, Hadlock TD, Levine R (2004). A comparison of web and mail survey response rates. Public Opin Q.

[B43] Oudejans S, Schippers GM, Spits M (2006). Benchmark Leefstijltraining in de Verslavingszorg.

[B44] Schafer JL, Graham JW (2002). Missing data: our view of the state of the art. Psychol Methods.

[B45] van Buuren S, Oudshoorn CGM (1999). Flexible multivariate imputation by MICE.

[B46] Cohen J (1988). Statistical power analysis for the behavioral sciences.

[B47] Dolan P (1997). Modeling valuations for the EuroQol health states. Med Care.

[B48] Open Health Measures. http://www.openhealthmeasures.org.

[B49] Whang W, Sisk JE, Heitjan DF, Moskowitz AJ (1999). Probabilistic sensitivity analysis in cost-effectiveness. an application from a study of vaccination against pneumococcal bacteremia in the elderly. Int J Technol Assess Health Care.

[B50] Briggs AH, Wonderling DE, Mooney CZ (1997). Pulling cost-effectiveness analysis up by its bootstraps: a non-parametric approach to confidence interval estimation. Health Econ.

[B51] Barber JA, Thompson SG (2000). Analysis of cost data in randomized trials: an application of the non-parametric bootstrap. Stat Med.

[B52] Eysenbach G (2005). The law of attrition. J Med Internet Res.

[B53] Langenbucher J, Merrill J (2001). The validity of self-reported cost events by substance abusers: limits, liabilities and future directions. Eval Rev.

[B54] Del Boca FK, Darkes J (2003). The validity of self-reports of alcohol consumption: state of the science and challenges for research. Addiction.

[B55] Sobell LC, Maisto SA, Sobell MB, Cooper AM (1979). Reliability of alcohol abusers' self-reports of drinking behavior. Behav Res Ther.

[B56] Tourangeau R, Steiger DM, Wilson D (2002). Self-administered questions by telephone: evaluating interactive voice responses. Public Opin Q.

[B57] Tabachnick B, Fidell L (2007). Using Multivariate Statistics.

[B58] Horton NJ, Kim E, Saitz R (2007). A cautionary note regarding count models of alcohol consumption in randomized controlled trials. BMC Med Res Methodol.

[B59] Hesterberg T, Moore DS, Monaghan S, Clipson A, Epstein R, Moore DS, McCabe GP (2005). Bootstrap Methods and Permutation Tests. Introduction to the Practice of Statistics.

